# Informing Global Cost-Effectiveness Thresholds Using Country Investment Decisions: Human Papillomavirus Vaccine Introductions in 2006-2018

**DOI:** 10.1016/j.jval.2020.07.012

**Published:** 2021-01

**Authors:** Mark Jit

**Affiliations:** 1 Department of Infectious Disease Epidemiology, Faculty of Epidemiology and Population Health, London School of Hygiene & Tropical Medicine, London, United Kingdom; 2 Modelling and Economics Unit, National Infections Service, Public Health England, London, United Kingdom; 3 School of Public Health, University of Hong Kong, Hong Kong SAR, China

**Keywords:** cost-effectiveness analysis, cost-effectiveness thresholds, human papillomavirus vaccination, incremental cost-effectiveness ratio

## Abstract

**Objectives:**

Cost-effectiveness analysis can guide decision making about health interventions, but the appropriate cost-effectiveness threshold to use is unclear in most countries. The World Health Organization (WHO) recommends vaccinating girls 9 to 14 years against human papillomavirus (HPV), but over half the world’s countries have not introduced it. This study aimed to investigate whether country-level decisions about HPV vaccine introduction are consistent with a particular cost-effectiveness threshold, and to estimate what that threshold may be.

**Methods:**

The cost-effectiveness of vaccinating 12-year-old girls was estimated in 179 countries using the Papillomavirus Rapid Interface for Modelling and Economics (PRIME) model, together with vaccine price data from World Health Organization’s Market Information for Access to Vaccines database. In each year from 2006 to 2018, countries were categorized based on (1) whether they had introduced HPV vaccination, and (2) whether the incremental cost-effectiveness ratio for HPV vaccine introduction fell below a certain cost-effectiveness threshold.

**Results:**

A cost-effectiveness threshold of 60% to 65% of GDP per capita has the best ability to discriminate countries that introduced vaccination, with a diagnostic odds ratio of about 7. For low-income countries the optimal threshold was lower, at 30% to 40% of GDP per capita.

**Conclusions:**

A cost-effectiveness threshold has some ability to discriminate between HPV vaccine introducer and non-introducer countries, although the average threshold is below the widely used threshold of 1 GDP per capita. These results help explain the current pattern of HPV vaccine use globally. They also inform the extent to which cost-effectiveness thresholds proposed in the literature reflect countries’ actual investment decisions.

## Highlights

• Most countries do not have cost-effectiveness thresholds; a threshold of 1 times gross domestic product per capita is often used despite lack of empirical support.
• A cost-effectiveness threshold of 60% to 65% of gross domestic product per capita (but less in low-income countries) has the best ability to discriminate countries that have introduced human papillomavirus vaccination.
• This may help explain why most countries have yet to introduce human papillomavirus vaccination despite World Health Organization recommendations and cost-effectiveness analyses suggesting they should do so.

## Introduction

Cost-effectiveness analysis has been promoted as a tool to help countries make good decisions about allocation of healthcare spending. Its use has greatly expanded in the last 2 decades, particularly in low- and middle-income countries.^1^

In its most common form to inform a decision about whether or not to make a particular health investment, the cost per quality-adjusted life-year gained or disability-adjusted life-year (DALY) averted for the investment is calculated incrementally to the next best alternative (eg, the status quo). This incremental cost-effectiveness ratio (ICER) is then compared to a cost-effectiveness threshold (CET). If the ICER is above the CET, then the investment is generally deemed not cost-effective, although other criteria like equity and acceptability are usually also recommended to be considered alongside cost-effectiveness.^2^

Analysts are hindered from this procedure, because only a few countries have publicly available data about the appropriate CET to use.^3^ In the absence of such information, one widely used CET is to spend a maximum of 1 to 3 times gross domestic product (GDP) per capita to avert a DALY.^4^ This CET was originally proposed by the World Health Organization (WHO)’s Commission on Macroeconomics and Health using human capital arguments around the market value created by averting a DALY. Nevertheless, this CET has been widely criticized for failing to account for healthcare budget limits and the opportunity cost of healthcare spending in most countries.^4^, ^5^, ^6^ Indeed, the WHO itself has cautioned against use of such a CET for country-level decision making.^2^ A previous review found that many countries had not introduced human papillomavirus (HPV) or rotavirus vaccines despite their being found to be cost-effective according to this CET.^5^

An alternative approach that has been suggested to estimate an appropriate CET is to consider the marginal productivity of current healthcare expenditure, because this represents the health gains that may be displaced by new investments. This approach was used to suggest that a CET of around £13 000 (below 50% of England’s GDP per capita) per quality-adjusted life-year gained should be used in England.^7^ This analysis required information on healthcare spending and disease-specific mortality across different program categories, but such data are generally not available in most countries. Nevertheless, simpler analyses using either changes in healthcare expenditure overall^8^ or extrapolation of the English analysis to other countries^9^ suggest that CETs for low- and middle-income countries should be set well below GDP per capita.

The link between these econometric analyses on national indicators and decisions about individual technologies still has to be established. Examining individual decisions at the country level to see what kind of CET they are consistent with (if any) would be useful to inform discussions around appropriate CETs. In particular, understanding decisions made by countries in different income categories and regions could bring understanding about the determinants of health opportunity costs in different countries. Nevertheless, such analyses would require information about both price and cost-effectiveness of the technology, which are rarely available across all countries.

One notable exception is HPV vaccination. The HPV vaccine was first licensed in the United States in 2006. Vaccine prices are tiered at levels that would theoretically allow countries at all income levels to purchase it. Prices that countries pay to procure the vaccine have been collected by the WHO since 2013 based on anonymous country reporting.^10^ There is now strong evidence about the vaccine’s efficacy,^11^ health impact,^12^ and economic benefits, including a global cost-effectiveness evaluation that published ICERs for 179 countries.^13^ Vaccinating 9- to 14-year-old girls is recommended by the WHO in all countries,^14^ and indeed such a strategy forms an essential component of the WHO Director-General’s call for elimination of cervical cancer as a public health problem globally. The WHO directives were partly driven by cost-effectiveness considerations, based on a CET of 1 times GDP per capita.^15^ HPV vaccination has been adopted in many low-, middle-, and high-income countries, but over half the countries in the world have yet to introduce it.^16^

The purpose of this article is to investigate whether country-level decisions to introduce (or not introduce) HPV vaccination are consistent with a CET, and to determine what that CET may be.

## Methods

### Data Sources

#### Country categories

Historical classification of countries by income (low, upper middle, lower middle, and high) in 2006-2019 was obtained from the World Bank (http://databank.worldbank.org/data/download/site-content/OGHIST.xls). Categorization of countries into geographical regions (Africa, Americas, Eastern Mediterranean, Europe, Southeast Asia, and Western Pacific) was obtained from the WHO (https://www.who.int/choice/demography/mortality_strata/en/).

#### Cost-effectiveness

The cost-effectiveness of HPV vaccine introduction for 12-year old girls was estimated using the Papillomavirus Rapid Interface for Modelling and Economics (PRIME). PRIME is a static model of HPV vaccination that uses proportional impact to estimate the cost-effectiveness of HPV vaccination before sexual debut. Cost-effectiveness results for all countries have been publicly available since 2014^13^; the model itself is publicly available with a user-friendly Excel interface (http://primetool.org). In this analysis, the published results from 2014 were used (consistent with the online tool), apart from updating vaccine prices to reflect new data and assuming a 2-dose schedule was used instead of a 3-dose schedule from 2015 following revised WHO recommendations.^14^ The cost-effectiveness calculations account for costs of procuring and delivering 2 to 3 doses, costs averted by vaccination from avoiding cervical cancer treatment, and DALYs averted by vaccination from preventing morbidity and mortality owing to cervical cancer. Discounting at 3% for costs and benefits, a lifetime time horizon and healthcare perspective were used.

#### Year of vaccine introduction

The nonprofit organization PATH tracks HPV vaccine introduction status and year of introduction for all countries^16^ (data on file from D. Scott LaMontagne, personal communication). The first year of a program being initiated was regarded as the year of introduction, regardless of whether it was a national, subnational, pilot, or demonstration program. In a sensitivity analysis, only actual national long-term introductions were considered.

#### Cost of vaccine purchase

The WHO’s Market Information for Access to Vaccines database contains information on vaccine prices paid by reporting countries from 2013 to 2018.^10^ The exact country names are anonymized owing to commercial sensitivities; however, information is available about country characteristics including World Bank income category and WHO region. All data on HPV vaccine procurement prices were extracted from the database. When these data were disaggregated by year of procurement, World Bank income category, WHO region, and vaccine brand (Gardasil or Cervarix), no consistent pattern was observable (see Appendix 1 in Supplemental Materials found at https://doi.org/10.1016/j.jval.2020.07.012). Hence, instead of extrapolating a linear trend to years before 2013, the average procurement cost for each World Bank income category and WHO region was assigned to all countries within those categories regardless of year (see Table 1). In categories where there were no data, the average across the World Bank income category was used instead.

**Table 1:** Average price paid per HPV vaccine dose (in USD) by countries in WHO’s MI4A database from 2013-2018.

WHO region	World Bank country income classification			
	High	Upper-middle	Lower-middle	Low
Africa	27.87	15.25	4.60	4.59
Americas	10.75	9.35	7.18	No data
Eastern Mediterranean	No data	No data	No data	No data
Europe	48.86	50.04	4.75	No data
Southeast Asia	No data	9.59	5.73	No data
Western Pacific	56.31	20.17	8.31	No data

HPV indicates human papillomavirus; MI4A, Market Information for Access to Vaccines; WHO, World Health Organization.

#### Cost of vaccine delivery

Vaccine delivery was assumed to cost $5, $15, and $25 per fully immunized girl for low-income, middle-income, and high-income countries, respectively, based on previous analyses.^13^

### Analysis

The estimated ICER of HPV vaccine introduction was calculated for 179 countries. Different CETs were then generated, ranging from 0% to 100% (in 1% increments) of each country’s GDP per capita. In each year from 2006 to 2018, countries were categorized based on (1) whether they had already introduced HPV vaccination, and (2) whether the ICER of HPV vaccine introduction fell at or below each CET. For each year and CET, countries were then placed into 4 categories to determine the diagnostic ability of that CET in predicting country introduction status: (1) true positive (TP): vaccine introduced, ICER ≤ CET; (2) false positive (FP): vaccine not yet introduced, ICER ≤ CET; (3) true negative (TN): vaccine not yet introduced, ICER > CET; and (4) false negative (FN): vaccine introduced, ICER > CET.

For each year and CET value, 2 measures of the accuracy of the CET were then calculated: (1) diagnostic accuracy, (TP + TN) / (TP + FP + FP + TN), and (2) diagnostic odds ratio, (TP / FP) / (FN / TN). The diagnostic odds ratio captures the ability of the CET to discriminate between true positives (countries that introduced HPV vaccination) and true negatives (countries that did not introduce HPV vaccination).

Graphs of these measures by CET value were plotted by fitting cubic splines through the results using the R function smooth.spline. All analyses were conducted in R version 3.5.2.

## Results

**Figure 1:**
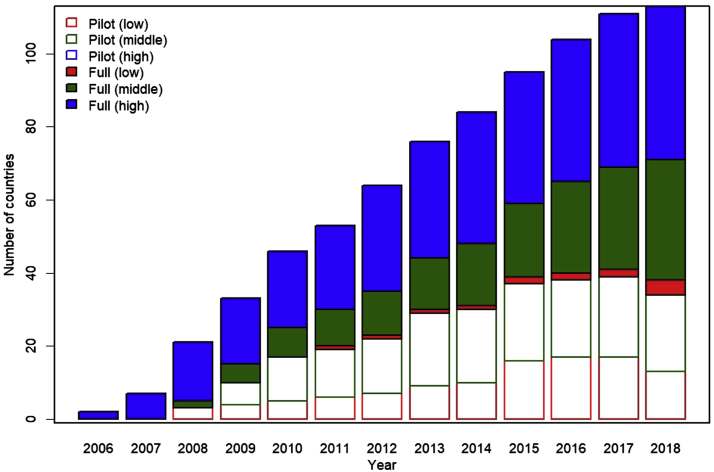
Number of countries each year with full or pilot introductions of HPV vaccination by income group.

Figure 1 shows the number of countries that had introduced HPV vaccination each year from 2006 to 2018. The number of full introductions in high- and middle-income countries increased steadily each year. Pilot introductions in low- and middle-income countries began increasing after 2008, but these proceeded to full introductions mainly in middle-income countries. The number of pilot introductions in these increased until 2016, after which it plateaued.

HPV, human papillomavirus.

Countries having introduced HPV vaccination had on average higher ICERs than the non-introducers until 2012. After 2012, ICERs for introducers dropped below that in non-introducers as more middle-income countries started to introduce vaccination. In 2019, vaccine introducers had a median ICER of 15.21% of GDP per capita (interquartile range 8.34-26.94) compared to 21.48% (9.97-52.02) for non-introducers (see Appendix 2 in Supplemental Materials found at https://doi.org/10.1016/j.jval.2020.07.012 for further details).

**Figure 2:**
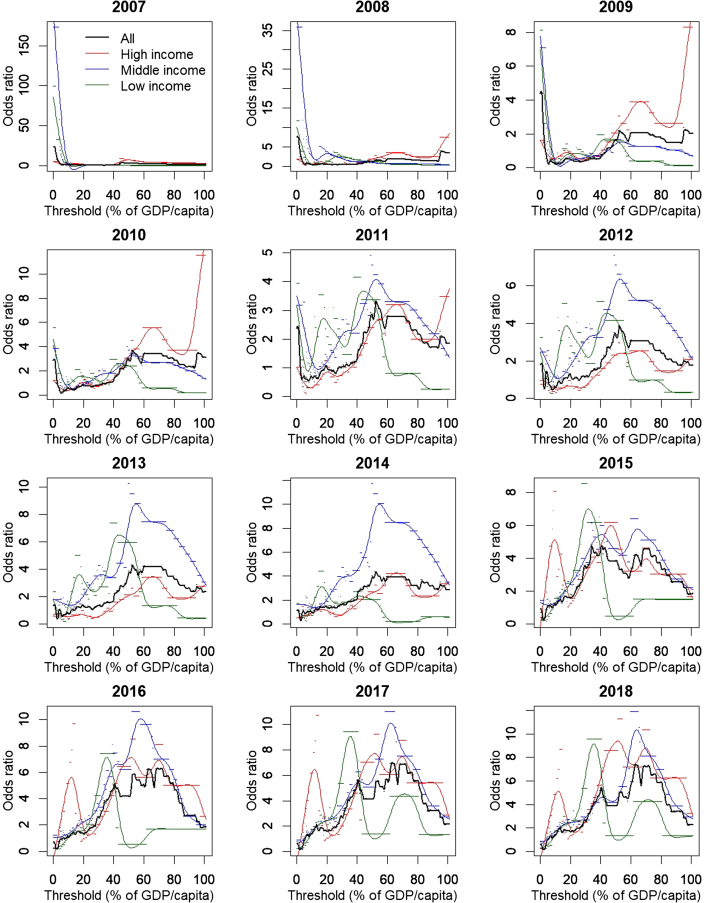
Diagnostic odds ratio of different cost-effectiveness thresholds in predicting HPV vaccine introductions. Dots indicate actual model results while lines are cubic splines fitted to the dots.

GDP, gross domestic product; HPV, human papillomavirus.

Figure 2 shows the diagnostic odds ratios for different CET values; corresponding diagnostic accuracy values are shown in Appendix 3 in Supplemental Materials found at https://doi.org/10.1016/j.jval.2020.07.012. Before 2011, any CET has poor diagnostic ability since few countries had introduced HPV vaccination regardless of how cost-effective it was. Over time, an appropriately chosen CET performed increasingly well in discriminating vaccine introducers and non-introducers, reaching an odds ratio of around 7 by 2018 at a CET of 60% to 65% of GDP per capita. At that CET, diagnostic accuracy could reach around 70%. Nevertheless, in low-income countries, a CET of only 30% to 40% of GDP per capita has the best discriminatory power. Restricting positives to full introductions only still gave an optimum CET at 60% to 65% of GDP per capita, but the diagnostic odds ratio and accuracy were lower.

## Conclusions

This study investigated the extent to which cost-effectiveness at different CETs influenced HPV vaccine introduction decisions by linking the cost-effectiveness of introducing HPV vaccination for 12-year-old girls with country vaccine introduction status. Results suggest that a CET of 60% to 65% of GDP per capita has moderate ability to discriminate between introducing and non-introducing countries, particularly in the later years of the period from 2006 to 2018. This optimum CET had a diagnostic odds ratio of about 7, meaning that the odds of the ICER being below the CET in introducers to non-introducers is about 7 times the odds of being above the CET.

The corresponding diagnostic accuracy of the optimum CET is around 65%, so about a third of countries made vaccine introduction decisions (either positive or negative) that were not predicted by the CET. Together, the diagnostic accuracy and odds ratio suggest that cost-effectiveness had an important influence in introduction decisions, but was not the only consideration. This is consistent with advice from WHO^2^ and others^17^ that cost-effectiveness should be considered as one input in a multi-criteria decision framework alongside other evidence-based criteria such as affordability, sustainability, equity, and acceptability. Also, the ability of the optimum CET to predict country decisions in most cases does not imply that cost-effectiveness was formally considered in decision making. Even if it was, the assumptions and input parameters used in a country’s own evaluation may have differed from those used in this analysis. It could instead indicate that key inputs to the ICER such as high cervical cancer burden compared with the cost of vaccination were influential in the decision. Nevertheless, the pattern of country decisions does imply that the level of disease burden and vaccine costs that was considered acceptable in each country was on average consistent with a CET of 60% to 65% of GDP per capita.

The most discriminatory CET was lower than the widely used 1-to-3-times-GDP-per-capita threshold, and for low-income countries is similar to CETs suggested in econometric analyses of healthcare spending.^8^^,^^9^ For middle-income countries it is generally higher than those in the econometric analyses. Nevertheless, the ICERs estimated in this study may be underestimates, because they rely on the simple PRIME model that ignores some vaccine benefits such as herd effects (indirect protection of non-vaccinees) and reduction in non-cervical disease (such as vulvar, vaginal, penile, anal, and oropharyngeal cancer as well as anogenital warts) to be able to generate results for almost all countries in the world. Incorporating these benefits may bring the optimum CET to close to the levels suggested in previous econometric analyses.

The analysis did not distinguish between sources of financing that were internal or external to the country. Vaccine introduction costs in low-income countries are heavily subsidized by Gavi, the Vaccine Alliance. Hence from a country’s perspective the ICER of vaccine introduction is even lower than suggested here, so the optimum CET is also lower.

The analyses were conducted for each year from 2006 (when HPV vaccination was first licensed in the United States) to 2019. World Bank country income category and HPV vaccine introduction status were updated annually. Nevertheless, other parameters (including GDP per capita, disease burden, vaccine costs, and treatment costs) were not updated. This was for several reasons. Firstly, data on treatment costs and cervical cancer burden are not updated annually in most countries. For instance, vaccine price data only started to be collected in 2014, while cervical cancer burden estimates are only updated once every few years by the International Agency for Research on Cancer.^18^ Furthermore, countries themselves do not generally have access to any more recent data than these on which to base introduction decisions, so the decisions themselves are unlikely to reflect annually updated parameters. Over time, it is likely that both GDP per capita (and hence the absolute value of the corresponding CETs) and economic impact of cervical cancer will increase for most countries, but it is difficult to determine which of these trends is more important without much better data.

HPV vaccination of young adolescent girls has been found to be cost-effective in almost every country using a CET of 1 GDP per capita.^13^ Yet most countries (particularly low-income countries) have yet to introduce vaccination despite WHO recommendations to do so. Obstacles to introduction include misunderstandings about vaccine safety and efficacy, difficulty in delivering 2 doses of the vaccine to adolescents, as well as competition with other vaccines that are also recommended for introduction (such as rotavirus and pneumococcal conjugate vaccines).^19^ Some of these obstacles suggest that the health opportunity costs of HPV vaccine introduction may be considerable in terms of the displaced human, planning, and financial resources in generating health. This is consistent with our observation that the CET that best predicts country introduction decisions is well below 1 GDP per capita, particularly in low-income countries. This suggests that some countries, particularly low-income countries, may need additional financial and planning resources for HPV vaccine introductions to become universal and the WHO’s goal of cervical cancer elimination to be achieved.

## Supplemental Material

Appendices 1-3
