# Supplementary material for: Informing Global Cost-Effectiveness Thresholds Using Country Investment Decisions: Human Papillomavirus Vaccine Introductions in 2006-2018
Source: Value Health. 2021 Jan;24(1):61–6. doi: 10.1016/j.jval.2020.07.012 (PMC7813214; doi:10.1016/j.jval.2020.07.012)

Appendices to " Informing global cost-effectiveness thresholds using country investment decisions: human papillomavirus vaccine introductions in 2006-2018"

Contents

Appendix 1. Price paid for each dose of HPV vaccine. Dots show price averaged across countries while lines show the best fitting linear trendline. 2

Appendix 2. Incremental cost-effectiveness ratio (ICER) of HPV vaccine introduction for countries which had and had not introduced HPV vaccine by a certain year. 3

Appendix 3. Diagnostic accuracy of different cost-effectiveness thresholds in predicting HPV vaccine introductions. Dots indicate actual model results while lines are cubic splines fitted to the dots. 4

Appendix 1. Price paid for each dose of HPV vaccine. Dots show price averaged across countries while lines show the best fitting linear trendline. Results are stratified by vaccine brand (Gardasil, Cervarix), World Bank income group (high-income countries HIC, upper middle-income countries UMIC, lower middle-income countries LMIC and low-income countries LIC) and WHO region (Africa AFR, Americas AMR, Eastern Mediterranean EMR, Europe EUR, South-East Asia SEAR, Western Pacific WPR).

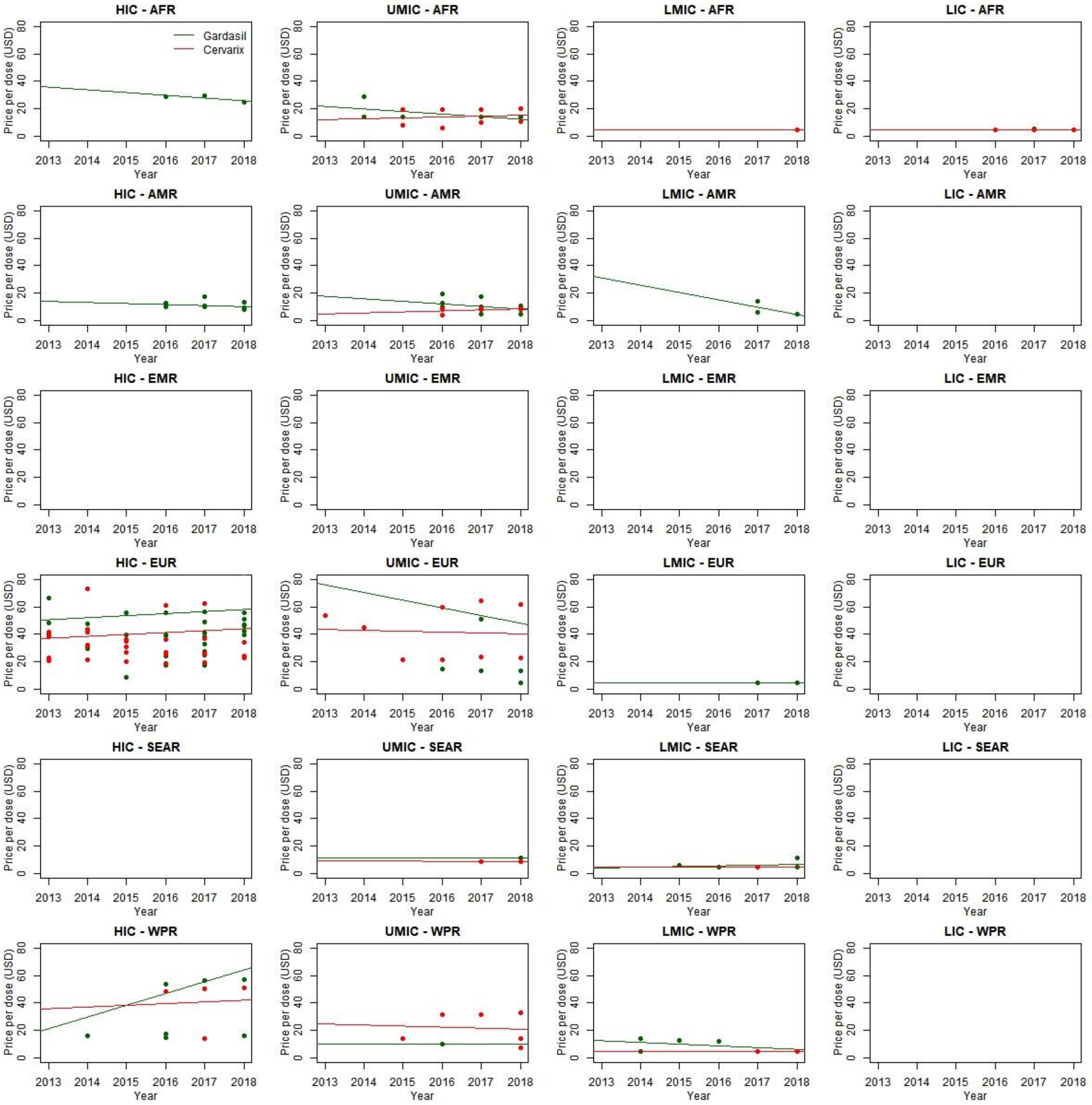

Appendix 2. Incremental cost-effectiveness ratio (ICER) in cost per DALY averted as % of GDP per capita of HPV vaccine introduction for countries which had and had not introduced HPV vaccine by a certain year.

Vaccine introducers

	ICER quantile (% of GDP per capita)				
	0	0.25	0.5	0.75	1
2006	6.62	13.7	20.8	27.8	34.9
2007	6.62	23.1	39.8	41.9	44
2008	5.88	15.4	31.6	43.6	94.9
2009	5.88	16.2	31.6	41.9	304
2010	5.88	14.7	25.3	39.2	304
2011	4.07	11.7	22.2	37.5	304
2012	3.56	11.7	20.5	35	304
2013	3.56	11.5	19.6	35	304
2014	3.56	11.4	19.6	35	304
2015	2.81	8.27	15.2	25.4	239
2016	2.81	8.18	15.3	26.7	239
2017	2.81	8.07	15.1	26.7	239
2018	2.81	8.34	15.2	26.7	239

Vaccine non-introducers

	ICER quantile (% of GDP per capita)				
	0	0.25	0.5	0.75	1
2006	1.79	9.42	18.9	33.3	304
2007	1.79	9.77	18.9	32.9	304
2008	1.79	10	18.9	34.7	304
2009	1.79	9.5	18.9	36.2	303
2010	1.79	9.95	20.3	46.6	303
2011	1.79	10.4	21.1	46.9	287
2012	1.79	10.3	21.3	53.5	313
2013	1.79	10.8	23.1	56	313
2014	1.79	11.3	25	56.5	313
2015	1.18	8.47	19.6	42.8	229
2016	1.18	8.37	17.9	43.6	229
2017	1.18	9.62	20.6	46.6	229
2018	1.18	9.97	21.5	52	229

Appendix 3. Diagnostic accuracy of different cost-effectiveness thresholds in predicting HPV vaccine introductions. Dots indicate actual model results while lines are cubic splines fitted to the dots.

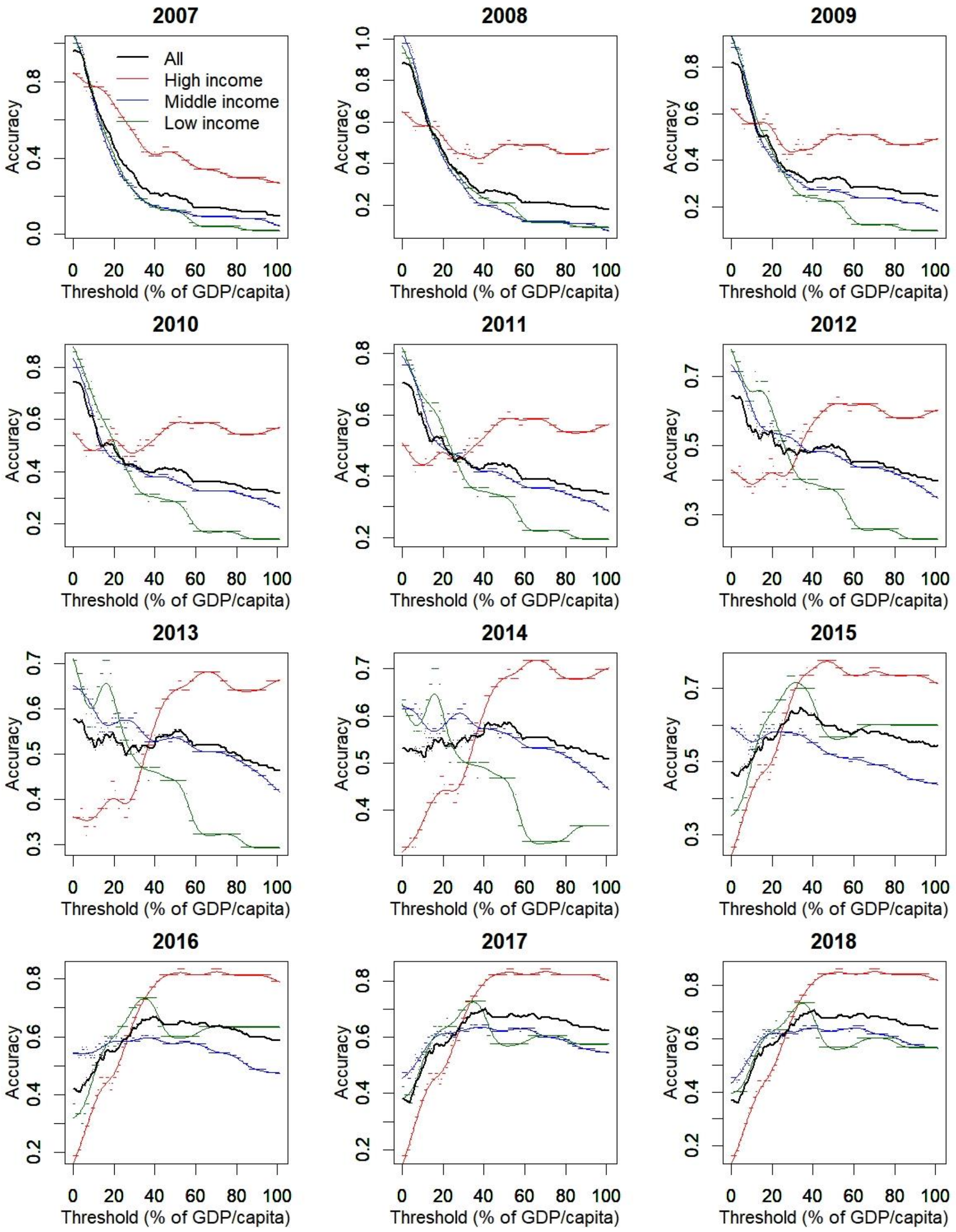

Supplement: Appendices 1-3 [file mmc1.pdf]
